# A longitudinal study on the effect of aerobic exercise intervention on the inhibitory control in college students with internet addiction

**DOI:** 10.3389/fnhum.2025.1500399

**Published:** 2025-02-26

**Authors:** Yi Wang, Xiangkun Li

**Affiliations:** ^1^School of Physical Education, Henan Polytechnic University, Jiaozuo, Henan, China; ^2^School of Sports Science, Jishou University, Jishou, Hunan, China

**Keywords:** aerobic exercise, internet addiction, reactive inhibitory control, time-frequency analysis, college students

## Abstract

**Objectives:**

This study aimed to investigate the effects of aerobic exercise on reactive inhibitory control in college students with internet addiction, examining both behavioral and electrophysiological changes over time.

**Methods:**

A longitudinal study design was adopted, involving 48 male college students with internet addiction who were randomly assigned to either a control group or an experimental group. Participants in the experimental group engaged in 40 min aerobic cycling sessions three times per week for 12 weeks, while the control group maintained their usual physical activity levels without any intervention. A 3 × 2 × 3 mixed-factorial design was utilized, incorporating three time points (pre-experiment, 6 and 12 weeks), two groups (control and experimental), and three electrode sites (Fz, F3, F4). This design enabled the examination of the effects of aerobic exercise on reactive inhibitory control and its temporal dynamics in college students with internet addiction.

**Results:**

A significant main effect of group was observed. Specifically, the experimental group demonstrated a significantly higher Nogo accuracy rate compared to the control group at both the mid-test (*P* < 0.01) and post-test (*P* < 0.001). Within the experimental group, the Nogo accuracy rate at the mid-test and post-test was significantly higher than at the pre-test (*P* < 0.001), with the post-test accuracy rate also significantly higher than the mid-test (P < 0.05). Time-frequency analysis revealed that, under the Nogo task, the energy values in the beta frequency band during the early (100–500 ms) and late (600–750 ms) time windows were significantly higher at the mid-test and post-test compared to the pre-test (*P* < 0.05), with the post-test values significantly exceeding those at the mid-test (*P* < 0.05).

**Conclusion:**

(1) Moderate-intensity aerobic exercise significantly improves reactive inhibitory control in college students with internet addiction, with the magnitude of improvement increasing over the duration of the intervention. (2) Increased beta band energy during the early (100–500 ms) and late (600–750 ms) time windows serve as a key neurophysiological indicator of this enhancement.

## 1 Introduction

Internet addiction (IA), also known as problematic internet use (PIU), is a behavioral addiction characterized by psychological dependence on various internet applications, such as online social networking, gambling, gaming, cybersex, and e-shopping (Nasser et al., 2020; Sharifat and Suppiah, 2021; Wang et al., 2021). It is marked by uncontrolled internet usage and an intense craving for online activities (Wang et al., 2019). IA increases the risk of impairments in psychological and social functioning, leading to issues such as depression, anxiety, and difficulties in academic performance, work responsibilities, and interpersonal relationships (Choi et al., 2014).

Inhibitory control refers to the ability to suppress or restrain thoughts or behaviors that are unrelated to the current task but exert a dominant influence (Garavan et al., 1999). Impairments in inhibitory control can lead to the breakdown of behavioral regulation, often manifesting as impulsive behaviors. In laboratory settings, inhibitory control is typically divided into motor inhibition and interference inhibition (Wostmann et al., 2013). Motor inhibition refers to the ability to suppress a preplanned motor response and is commonly assessed using tasks such as the Go/Nogo or stop-signal paradigms (Donders, 1969; Logan et al., 1984). In contrast, interference inhibition involves the ability to resolve response conflicts caused by irrelevant but incompatible stimuli. This type of inhibitory control is typically studied using the Simon (Simon and Rudell, 1967), Eriksen flanker (Davranche et al., 2009), and Stroop tasks (Zajano et al., 1981).

Motor inhibition is a complex construct comprising at least two distinct neuropsychological domains: (1) reactive inhibition, which refers to the ability to immediately halt a response upon receiving a stop instruction, and (2) proactive inhibition, which involves adjusting motor strategies based on contextual cues (Mirabella, 2021). This study utilized the Go/Nogo task, a widely recognized behavioral paradigm for examining the effects of addictive behaviors on reactive inhibition. Within this framework, reactive inhibition is assessed using critical metrics, including reaction time (e.g., response latency) and accuracy measures (e.g., hit rates and false alarm rates), which are essential for evaluating inhibitory control effectiveness (Ding et al., 2014; Dong et al., 2010; Verdejo-Garcia et al., 2007). Therefore, our research focused primarily on reactive inhibition performance in college students with IA. All subsequent discussions and interpretations of the experimental findings are framed within this context.

Empirical research indicates that individuals with IA are more prone to distractions during cognitive tasks compared to non-addicted individuals and exhibit reduced error monitoring capabilities and impaired inhibitory control (Dong et al., 2010; Littel et al., 2012; Mathews et al., 2005). Studies on the neurological underpinnings of addiction have shown that abnormalities in inhibitory control are closely linked to addictive mechanisms. Enhancing inhibitory control in individuals with addiction may represent a critical intervention for modifying addictive behaviors (Ceceli et al., 2022; Kuss and Griffiths, 2012).

Research on physical exercise interventions in individuals with substance addiction has shown that aerobic exercise can stimulate dopamine secretion in the brain. Dopamine, a key neurotransmitter, is transmitted through the basal ganglia, originating from the substantia nigra and ventral tegmental area and projecting to the striatum. From the striatum, it travels through the frontal-striatal and cortical-striatal circuits, which include connections between the striatum, prefrontal cortex, and motor cortex (Alexander and Crutcher, 1990). These circuits play a critical role in transmitting dopamine signals to the prefrontal and motor cortices, reactivating the brain’s system to restore normal functioning (Petzinger et al., 2015). This suggests that aerobic exercise may restore impaired cognitive functions in individuals with addiction. However, current research on the effects of aerobic exercise on inhibitory control primarily focuses on individuals with substance addiction (Wang et al., 2015; Wang et al., 2016; Zhu et al., 2023). Although emerging studies have explored the impact of aerobic exercise on inhibitory control in individuals with IA, these investigations have been limited to acute exercise sessions (Fan et al., 2021; Zhou and Wang, 2022). Thus, the long-term effects of aerobic exercise on inhibitory control in individuals with non-substance addiction remain unclear.

When performing cognitive tasks, participants generate event-related brain potentials (ERPs) in response to stimuli and modulate transient neural oscillatory rhythms, which provide rich information about cognitive processing (Wang et al., 2023; Weise et al., 2023). Understanding these oscillatory activities is crucial for elucidating the neural mechanisms through which exercise influences cognitive functions (Tanigawa et al., 2022). Against this backdrop, the present study combines the Go/Nogo task paradigm with ERP recordings, employing a longitudinal research design and time-frequency analysis to investigate the effects of moderate-intensity aerobic exercise on inhibitory control in university students with IA and to track changes over the intervention period. This study aims to provide empirical evidence for the effectiveness of aerobic exercise in enhancing inhibitory control among university students with IA, thereby offering a theoretical and practical foundation for universities to develop comprehensive extracurricular physical activity programs aimed at improving inhibitory control in these students.

Previous research has demonstrated that the beta band in the frontal lobe is closely associated with inhibitory control functions. For example, Alegre et al. (2004) found that frontal and central oscillatory changes in the beta band are related to different aspects of the motor process in Go/Nogo paradigms. Miller and Cohen (2001) proposed an integrative theory suggesting that the prefrontal cortex, where beta band activity is prominent, plays a critical role in inhibitory control. Similarly, Wagner et al. (2018) and Schaum et al. (2021) emphasized the importance of the beta band in inhibitory control processes. Notably, studies by Aron et al. (2014) 2015 have shown that specific regions in the prefrontal cortex are highly specialized for executive functions, with inhibitory control being a key component. Their research provides strong evidence that the prefrontal cortex is a critical region for regulating inhibitory processes. These findings suggest that power changes in the frontal lobe beta band during Go/Nogo tasks are associated with reactive inhibition functions, reflecting the processes involved in inhibiting motor responses.

Therefore, this study hypothesizes that moderate-intensity aerobic exercise can significantly improve inhibitory function in college students with IA, with the improvement becoming more pronounced as the duration of the exercise intervention increases. Furthermore, an increase in beta band energy serves as a key neurophysiological indicator of this enhancement.

## 2 Methodology

### 2.1 Participants

The sample size for this study was determined using G*Power 3.1 software, based on the effect sizes of aerobic exercise interventions on inhibitory control reported in previous studies (de Greeff et al., 2018; Gusatovic et al., 2022; Li et al., 2024). Assuming a medium effect size (*f* = 0.25), a significance level of α = 0.05, and a desired power of 1 - β = 0.90, the software calculated that a minimum of 36 participants were required, equally distributed between two groups. To account for potential dropouts and adherence issues, the sample size was increased by 20%, resulting in a minimum of 44 participants, with 22 in each group. Recruitment targeted full-time male undergraduate students in their third year at a specific university, focusing on those with moderate IA. The inclusion criteria were as follows: (1) Moderate IA, defined by scores ranging from 60 to 79 on the Young Internet Addiction Scale; (2) Non-athlete status; (3) A Body Mass Index (BMI) between 18.50 and 24.99; (4) Responses to exercise contraindication questions that met the study’s experimental requirements; (5) Negative responses to all items on the Physical Activity Readiness Questionnaire (PAR-Q), ensuring participant safety during exercise (Pontifex et al., 2009); (6) An energy expenditure of less than 600 MET-min/week, as measured by the short form of the International Physical Activity Questionnaire (IPAQ), indicating a low level of physical activity (Macfarlane et al., 2007); (7) Normal or corrected vision, hearing, and color vision, with no history of cardio-cerebrovascular or mental illnesses. A total of 48 participants were recruited and randomly assigned to either the control group or the experimental group, with 24 participants in each group. During the study, data from five participants—four from the experimental group and one from the control group—were excluded due to attendance below 90% or withdrawal, resulting in the analysis of data from 43 participants. Independent sample *t*-tests were conducted to compare age, IA-related metrics, and health indicators between the two groups, revealing no significant differences (*P* > 0.05), as shown in Table 1. The study received ethical approval from the Ethics Committee of Henan ** University (Ethics Approval Number: 202305012).

**TABLE 1:** Baseline characteristics of participants mean M(SD).

Variable		Experimental group (*n* = 20)	Control group (*n* = 23)	t	*P*
Age/years	–	22.32 (1.74)	22.27 (0.75)	0.59	0.57
Data related to IA	Duration of internet Use	9.31 (2.42)	10.01 (3.25)	−1.53	0.33
	Score of IA	71.35 (4.23)	70.22 (3.23)	1.28	0.35
Health-related Data	BMI (kg/cm^2^)	21.51 (1.32)	22.22 (1.56)	−0.81	0.41
	Resting heart rate	69.41 (5.61)	68.65 (4.27)	0.66	0.52
	Energy expenditure (MET-minutes/week)	431.79 (112.61)	451.79 (131.28)	−0.97	0.38

### 2.2 Experimental design

This behavioral study employed a mixed-design experimental approach, incorporating two independent variables: three measurement time points (pre-test, mid-test, post-test) and two group conditions (control group, experimental group). The dependent variables were the accuracy rate and response time in task performance. Similarly, the ERP study utilized a mixed-design with an additional factor, using the energy values across different frequency bands as dependent variables. This design included three factors: three measurement time points (pre-test, mid-test, post-test), two group conditions (control group, experimental group), and three electrode sites (Fz, F3, F4).

### 2.3 Aerobic exercise protocol

The intervention in this study consisted of moderate-intensity stationary cycling. A VO_2max_ test was conducted 1 week before the intervention to determine individual exercise intensity levels. Following the “Chinese Consensus on Exercise Prescription (2023)” and considering the participants’ health profiles, an intensity of 55–60% of VO_2max_ was set as the moderate exercise intensity (Li et al., 2024). The corresponding heart rate, identified during the VO_2max_ test, served as the target heart rate for the aerobic exercise sessions. Each session lasted 40 min and was performed three times per week. After the 4th and 8th weeks, the experimental group underwent additional VO_2max_ tests to recalibrate the target heart rate at 55–60% of VO_2max_ intensity for the subsequent 4 weeks intervals (weeks 5 to 8 and 9 to 12). The total duration of the intervention was 12 weeks. Throughout the exercise period, participants wore a heart rate telemetry monitor (model: RCX3) to monitor their heart rates in real-time, ensuring strict adherence to the prescribed exercise intensity. As participants’ exercise capacity improved, the resistance on the stationary bike was adjusted to maintain the target heart rate intensity while increasing the workload.

### 2.4 Experimental task

**FIGURE 1:**
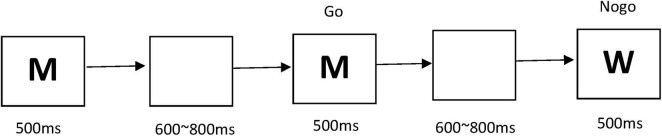
Go/Nogo task flowchart.

This study utilized the Go/Nogo paradigm. After pressing the “start” button, participants were presented with a sequence of letters, such as “M, M, W, M…” on the computer screen. Participants were instructed to press the V key (Go) if the current letter matched the preceding one, whereas they were required to withhold responses (Nogo) when the letters differed, with Nogo trials accounting for 30% of the total. The experiment consisted of four blocks, each containing 50 trials, resulting in a total of 200 trials. The stimulus presentation duration per trial was set at 500 ms, followed by an inter-trial interval ranging from 600 to 800 ms of a blank screen. Participants were encouraged to complete the tasks with both speed and accuracy. For detailed depictions of the specific tasks, refer to Figure 1.

In this task, participants were presented with a sequence of letters on the computer screen. When the current letter matched the preceding one, they were required to press the V key (Go); for letters that differed from the previous one (Nogo), they were to refrain from pressing any key. The experiment consisted of four blocks, each with 50 trials, totaling 200 trials. The stimulus presentation duration per trial was 500 ms, followed by an inter-trial interval of 600–800 ms of a blank screen.

### 2.5 Experimental procedure

The experiment was conducted in the Sports Psychology Laboratory at Henan **University, a facility designed to minimize external interference, featuring light-proof and sound-proof conditions. The indoor environment was carefully controlled, with a temperature maintained between 22 and 24°C and humidity levels between 40 and 50%, to ensure consistency and reduce external influences during the experimental process. Participants were fully informed about the experimental procedures and provided written informed consent. The experimental group participated in a 40 min aerobic exercise regimen on a stationary bicycle, consisting of a 5 min warm-up, 30 min of moderate-intensity exercise, and a 5 min cool-down, conducted three times per week on Wednesdays, Fridays, and Sundays. Each session was scheduled from 19:00 to 19:40 and continued for 12 weeks. Meanwhile, the control group abstained from any form of exercise training during the corresponding time slots. Throughout the study, all participants were instructed to maintain their regular physical activity levels, refrain from taking additional nutritional supplements, and avoid the intake of psychoactive drugs.

Both groups underwent inhibitory control testing and concurrent electroencephalogram (EEG) recordings at three specific time points: pre-test (prior to the commencement of the experiment), mid-test (after 6 weeks), and post-test (after 12 weeks). To prevent acute exercise effects from confounding the results, inhibitory control testing and EEG recordings for both groups were conducted 24 h following the completion of the exercise session for the experimental group. After a 10 min rest period, participants’ resting heart rate was measured, followed by the inhibitory control testing and EEG recording. For further details regarding the experimental procedure, refer to Table 2.

**TABLE 2:** Experimental procedures overview.

	Experimental group	Control group
1	Pre-test for inhibition function	Pre-test for inhibition function
2	6 weeks moderate-intensity aerobic exercise intervention	Maintain existing physical activity habits, no additional intervention
3	Complete inhibition function task, record ERP simultaneously	Complete inhibition function task, record ERP simultaneously
4	12 weeks moderate-intensity aerobic exercise intervention	Maintain existing physical activity habits, no additional intervention
5	Complete inhibition function task, record ERP simultaneously	Complete inhibition function task, record ERP simultaneously

### 2.6 Data collection and processing

#### 2.6.1 Behavioral data collection and processing

Behavioral data were collected using E-prime 3.0 software, with key metrics focusing on reaction time and accuracy for both Go and Nogo trials. The initial data preprocessing involved importing the data into Excel and removing any data points that exceeded three standard deviations from the mean or had an accuracy rate below 50%. The reaction time and accuracy rates for Go and Nogo trials served as the dependent variables for a repeated measures ANOVA, conducted across three time points (pre-test, mid-test, post-test) and two groups (control group, experimental group). When the data violated Mauchly’s test of sphericity, the Greenhouse-Geisser correction was applied to adjust the degrees of freedom and *P*-values. If a significant interaction between time and group was detected, a simple effects analysis followed by Bonferroni *post-hoc* tests was performed. Statistical significance was set at *P* < 0.05.

### 2.6.2 ERP data collection and processing

Electroencephalogram (EEG) data were collected using Recorder software from Brain Products, Germany, with Ag/AgCl electrodes placed according to the extended international 10–20 system using a 64-channel electrode cap. Reference electrodes were positioned at the bilateral mastoids, while the horizontal electrooculogram (HEOG) was placed 1 cm lateral to the right eye, and the vertical electrooculogram (VEOG) was positioned 1 cm above the left eye orbit. Impedance levels between the scalp and electrodes were maintained below 10 kΩ throughout the experiment to ensure signal integrity. EEG signals were continuously recorded after amplification, with a sampling rate of 1,000 Hz per channel.

Data processing was carried out using BP Vision-Analyzer software. Initially, the average potential of the bilateral mastoid electrodes, TP9 and TP10, was used as the reference, and the FCz electrode was re-referenced. A band-pass filter was applied with a low-pass filter set at 30 Hz, a high-pass filter at 0.1 Hz, and a slope of 24 dB/octave to eliminate power line noise and other artifacts. Independent component analysis (ICA) was employed to further reduce ocular artifacts. The data were segmented from 200 ms pre-stimulus to 800 ms post-stimulus, with the mean value during the 200 ms pre-stimulus period used for baseline correction. Trials with amplitudes exceeding ± 100 μV were marked as artifacts and were excluded from subsequent analysis.

### 2.7 Time-frequency analysis

The preprocessed EEG data were transformed into the time-frequency domain using wavelet transform, with a time window ranging from -200 to 800 ms relative to stimulus onset. Frequency representations from 1 to 30 Hz were extracted at 1 Hz intervals. The wavelet transform produced energy values for individual trials across temporal and spectral dimensions. These values were averaged to obtain the time-frequency representation for each participant under each experimental condition. The energy values were normalized to the baseline period (-200 to 0 ms) using Z-scores.

Building on previous research that highlights the close association between neural activity in the beta frequency band of the frontal lobe and inhibitory control functions (Gassmann et al., 2022; Kujala et al., 2023; Wu et al., 2023), this study deliberately chose three critical electrode sites: the midline frontal Fz, the left prefrontal F3, and the right prefrontal F4. The beta frequency band of 13–20 Hz within the time intervals of 100–500 ms and 600–750 ms post-stimulus, as illustrated in Figures 3, 4, was selected as the key time window for analysis. A repeated measures ANOVA with a factorial design of three (time points: pre-experiment, 6 weeks later, 12 weeks later) × 2 (groups: control, experimental) × 3 (electrode sites: Fz, F3, F4) was utilized to evaluate the energy values at these sites. When data violated the assumption of sphericity as assessed by Mauchly’s test, the Greenhouse-Geisser correction was applied to adjust the degrees of freedom and *P*-values. For significant interactions, simple effects analyses were performed, followed by *post-hoc* tests using the Bonferroni correction to account for multiple comparisons. The threshold for statistical significance was set at *P* < 0.05.

## 3 Results

### 3.1 Behavioral data results

Repeated measures ANOVAs were conducted to assess the effects on Go accuracy, Go reaction time, Nogo accuracy, and Nogo reaction time, with the independent variables being three measurement time points (pre-test, mid-test, post-test) and two group conditions (control and experimental). The results, detailed in Table 3, revealed significant main effects of time on Nogo accuracy [*F*_(2,82)_ = 12.17, *P* < 0.001, *η^2^* = 0.40], group [*F*_(1,41)_ = 38.11, *P* < 0.001, *η^2^* = 0.57], and a significant interaction between time and group [*F*_(2,82)_ = 7.79, *P* < 0.01, *η^2^* = 0.21]. *Post hoc* simple effects analysis indicated no initial difference in Nogo accuracy between groups at the pre-test stage *(P* > 0.05), confirming the baseline equivalence of the participant cohorts. Notably, significant group differences were observed at the mid-test (*P* < 0.01) and post-test (*P* < 0.001), indicating that the aerobic exercise intervention over both 6 and 12 weeks significantly enhanced Nogo accuracy in the experimental group compared to the control group. Within the experimental group, Nogo accuracy showed significant variation across the three time points (*P* < 0.001), with scores at mid-test and post-test being significantly higher than pre-test levels (*P* < 0.001), and post-test scores being higher than those at mid-test (*P* < 0.05). In contrast, the control group exhibited no significant changes in Nogo accuracy across the time points (*P* > 0.05). For Go accuracy, Go reaction time, and Nogo reaction time, no significant main or interactive effects were detected for time or group.

**TABLE 3:** Descriptive statistics and repeated measures ANOVA of inhibition function behavioral data for the control and experimental groups at three time points M(SD).

Time points	Group	Go accuracy (%)	*F*	*P*	Go reaction Time (ms)	*F*	*P*	Nogo accuracy (%)	*F*	*P*	Nogo reaction time (ms)	*F*	*P*
Pre-test	Control group (*n* = 23)	93.23 (3.21)	–	–	366.10 (31.02)	–	–	77.52 (6.21)	–	–	370.12 (21.23)	–	–
	Experimental group (*n* = 20)	94.62 (3.21)	–	–	348.10 (21.05)	–	–	76.23 (5.21)	–	–	360.32 (34.03)	–	–
Mid-test	Control group (*n* = 23)	93.68 (3.13)	–	–	349.55 (31.45)	–	–	79.39 (5.22)	–	–	358.07 (25.14)	–	–
	Experimental group (*n* = 20)	95.15 (2.61)	–	–	360.58 (21.65)	–	–	83.61 (4.12)^**^^###^	–	–	370.97 (34.14)	–	–
Post-test	Control group (*n* = 23)	94.65 (2.88)	–	–	358.81 (27.34)	–	–	79.96 (4.36)	–	–	366.62 (25.19)	–	–
	Experimental group (*n* = 20)	95.38 (3.21)	–	–	355.41 (26.34)	–	–	86.56 (7.12)^***^^###^^▲^	–	–	354.62 (26.89)	–	–
Main effect of time points			0.28	0.57—	–	0.29	0.61	–	12.17	< 0.001	–	1.02	0.41
Main effect of groups			0.27	0.62	–	0.45	0.52	–	38.11	< 0.001	–	0.83	0.38
Interaction effect of time points × groups			1.57	0.21	–	1.57	0.22	–	7.79	< 0.01	–	1.28	0.27

Intergroup comparison: compared with the control group (**P < 0.01, ***P < 0.001). Intragroup comparison: compared with the pre-test results (###*P* < 0.001); compared with the mid-test results (▲*P* < 0.05).

**FIGURE 2:**
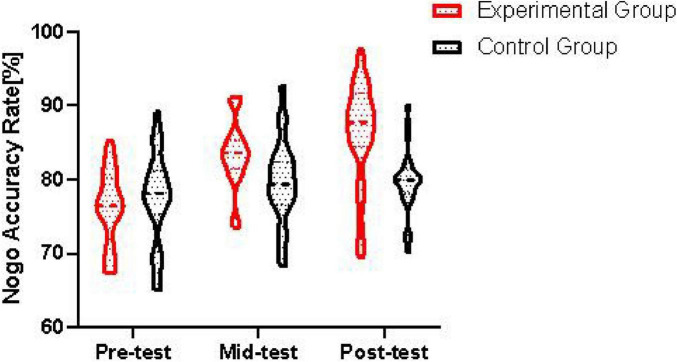
Changes in Nogo trial accuracy rates across intervention time points.

In conclusion, the behavioral data analysis reveals that moderate-intensity aerobic exercise significantly enhances the correct response rate in Nogo trials among college students with IA, with the beneficial effects intensifying over a longer intervention period. As depicted in Figure 2, the amelioration in inhibitory function attributable to moderate-intensity aerobic exercise follows the hierarchy of 12 weeks of exercise surpassing that of 6 weeks, which in turn exceeds the pre-experimental baseline.

The x-axis represents the three time points: pre-test, mid-test, and post-test. The y-axis shows the accuracy rate in Nogo trials. The data points and lines clearly demonstrate the changes in Nogo accuracy rate over time for both the control group and the experimental group. It can be seen that the experimental group shows a significant increase in Nogo accuracy rate from pre-test to mid-test and further to post-test, while the control group has relatively stable accuracy rates across the time points.

### 3.2 Time-frequency analysis results

#### 3.2.1 Beta band energy during go trials

**FIGURE 3:**
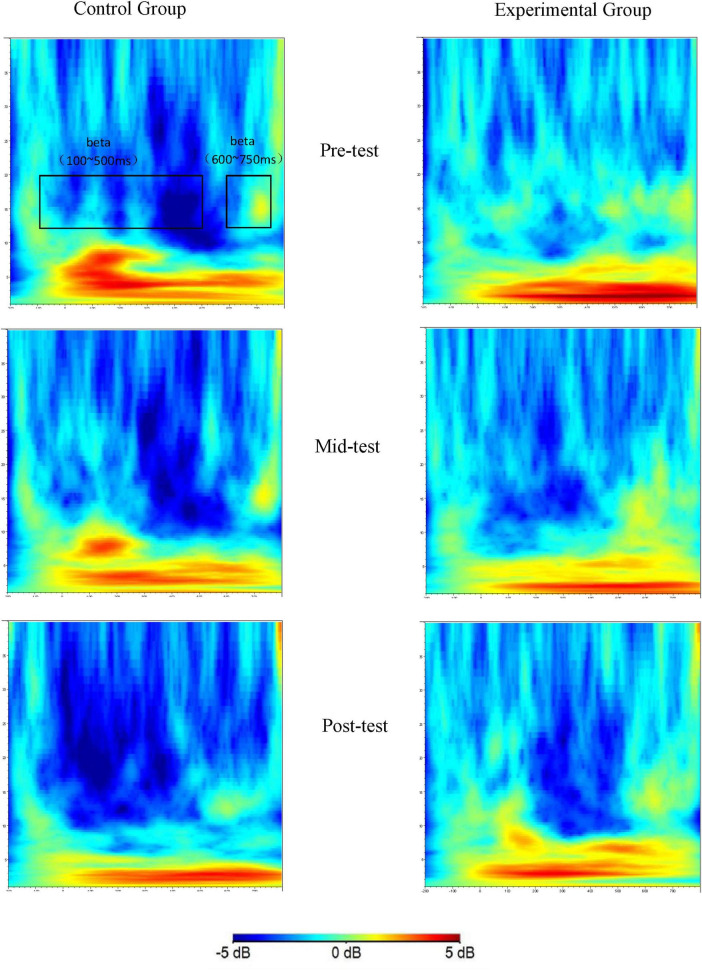
Time-frequency plots of the Go trial condition at three time points (Fz as an example).

Figure 3 shows the time-frequency distribution of beta band energy recorded at electrode Fz during Go trial conditions across the three time points of the study.

The time-frequency plots display the beta band energy values recorded at electrode Fz for the control group and the experimental group at the pre-test, mid-test, and post-test time points. The x-axis represents time relative to stimulus onset, ranging from -200 to 800 ms. The y-axis represents the frequency from 1 to 30 Hz. The color scale indicates the energy values in dB. Through these plots, the changes in beta band energy over time and frequency can be observed for both groups during the Go trials.

A repeated measures ANOVA was conducted to analyze the beta band energy values (100–500 ms) for both the experimental and control groups across the factors of three time points (pre-test, mid-test, post-test), two groups (control and experimental), and three electrode sites (Fz, F3, F4). As detailed in Table 4, the analysis revealed no significant main effects for time point (*P* > 0.05) or group (*P* > 0.05). However, a significant main effect was observed for electrode site [*F*_(2, 246)_ = 7.29, *P* < 0.01, *η^2^* = 0.27]. Additionally, no significant interactions were found between time point × group (*P* > 0.05), time point × electrode site (*P* > 0.05), group × electrode site (*P* > 0.05), or the three-way interaction of time point × group × electrode site (*P* > 0.05).

**TABLE 4:** Descriptive statistics and repeated measures ANOVA of beta (100∼500 ms) wave energy values at three time points under the Go condition M(SD).

Time points	Group	Electrode site	Energy value/dB	F	*P*
Pre-test	Control group (*n* = 23)	F3	−2.43 (0.31)	–	–
		Fz	−3.63 (0.12)	–	–
		F4	−1.92 (0.13)	–	–
	Experimental group (*n* = 20)	F3	−2.51 (0.35)	–	–
		Fz	−2.84 (0.21)	–	–
		F4	−1.89 (0.62)	–	–
Mid-test	Control group (*n* = 23)	F3	−2.38 (0.51)	–	–
		Fz	−3.17 (0.22)	–	–
		F4	−1.87 (0.15)	–	–
	Experimental group (*n* = 20)	F3	−2.48 (0.45)	–	–
		Fz	−2.70 (0.22)	–	–
		F4	−1.77 (0.44)	–	–
Post-test	Control group (*n* = 23)	F3	−2.39 (0.40)	–	–
		Fz	−3.52 (0.14)	–	–
		F4	−1.82 (0.39)	–	–
	Experimental group (*n* = 20)	F3	−2.51 (0.44)	–	–
		Fz	−2.63 (0.42)	–	–
		F4	−1.62 (0.56)	–	–
Main effect of time point				1.26	0.28
Main effect of group				1.04	0.31
Main effect of electrode site				7.29	< 0.01
Interaction effect of time point × group				0.85	0.37
Interaction effect of time point × electrode site				1.56	0.21
Interaction effect of group × electrode site				1.17	0.29
Three-way interaction effect of time point × group × electrode site				0.98	0.33

A repeated measures ANOVA was employed to evaluate the beta band energy values (600–750 ms) for both the experimental and control groups, factoring in three time points (pre-test, mid-test, post-test), two groups (control and experimental), and three electrode sites (Fz, F3, F4). The findings, as presented in Table 5, indicated no significant main effects for time point (*P* > 0.05) or group (*P* > 0.05). However, a significant main effect was identified for electrode site [*F*_(2, 246)_ = 4.33, *P* < 0.05, *η^2^* = 0.15]. Interactions between time point and group (*P* > 0.05), time point and electrode site (*P* > 0.05), group and electrode site (*P* > 0.05), and the three-way interaction of time point, group, and electrode site (*P* > 0.05) were all non-significant.

**TABLE 5:** Descriptive statistics and repeated measures ANOVA of beta (600∼750 ms) wave energy values at three time points under the Go condition M(SD).

Time points	Group	Electrode site	Energy value/dB	F	*P*
Pre-test	Control group (*n* = 23)	F3	−1.82 (0.31)	–	–
		Fz	−1.71 (0.20)	–	–
		F4	−1.92 (0.31)	–	–
	Experimental group (*n* = 20)	F3	−1.91 (0.24)	–	–
		Fz	−1.78 (0.51)	–	–
		F4	−1.79 (0.62)	–	–
Mid-test	Control group (*n* = 23)	F3	−1.78 (0.21)	–	–
		Fz	−1.66 (0.50)	–	–
		F4	−1.85 (0.23)	–	–
	Experimental group (*n* = 20)	F3	−1.88 (0.35)	–	–
		Fz	−1.58 (0.62)	–	–
		F4	−1.77 (0.54)	–	–
Post-test	Control group (*n* = 23)	F3	−1.69 (0.31)	–	–
		Fz	−1.41 (0.47)	–	–
		F4	−1.92 (0.59)	–	–
	Experimental group (*n* = 20)	F3	−1.82 (0.41)	–	–
		Fz	−1.48 (0.72)	–	–
		F4	−1.72 (0.46)	–	–
Main effect of time point				0.70	0.45
Main effect of group				0.41	0.57
Main effect of electrode site				4.33	0.03
Interaction effect of time point × group				0.88	0.31
Interaction effect of time point × electrode site				1.58	0.22
Interaction effect of group × electrode site				1.01	0.43
Three-way interaction effect of time point × group × electrode site				0.96	0.34

#### 3.2.2 Beta band energy during Nogo trials

**FIGURE 4:**
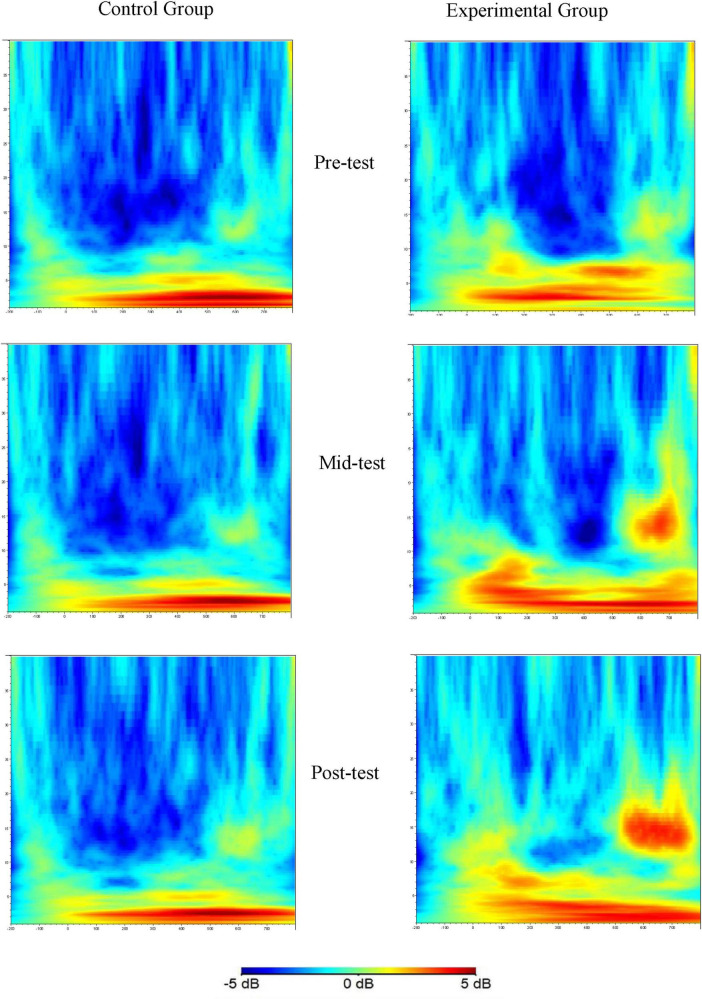
Time-frequency plots of the Nogo trial condition at three time points (Fz as an example).

Figure 4 illustrates the time-frequency distribution of beta band energy at electrode Fz during Nogo trial conditions across the three time points of the study.

These plots show the beta band energy values at electrode Fz for the control and experimental groups at different time points during Nogo trials. The x-axis, y-axis, and color scale have the same meanings as in Figure 3. The significant differences in beta band energy between the groups and across time points can be visualized, indicating the effects of aerobic exercise on the beta band energy associated with the Nogo task.

**FIGURE 5:**
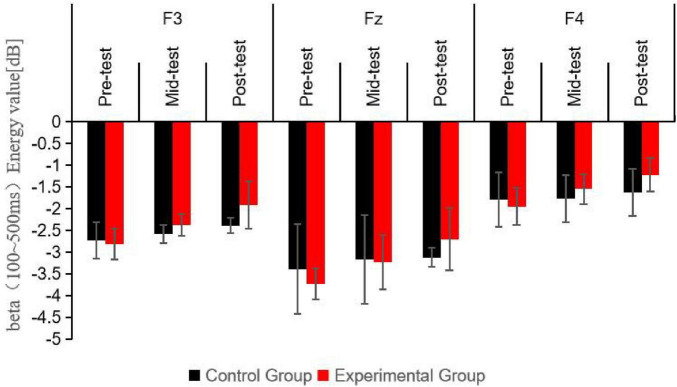
Schematic diagram comparing beta wave (100∼500 ms) energy values at different electrode sites for participants under the Nogo condition at three time points.

A repeated measures ANOVA was conducted to assess the beta band energy values (100–500 ms) for the experimental and control groups across three time points (pre-test, mid-test, post-test), two groups (control and experimental), and three electrode sites (Fz, F3, F4). The findings, as detailed in Table 6 and illustrated in Figure 5, indicated significant main effects for time point [*F*_(2, 123)_ = 11.52, *P* < 0.001, *η^2^* = 0.37], group [*F*_(1, 123)_ = 50.26, *P* < 0.001, *η^2^* = 0.66], and electrode site [*F*_(2, 246)_ = 9.78, *P* < 0.001, *η^2^* = 0.35]. Additionally, a significant interaction was observed between time point and group [*F*_(2, 123)_ = 8.52, *P* < 0.01, *η^2^* = 0.21]. However, no significant interactions were found between time point and electrode site, group and electrode site, or the three-way interaction of time point, group, and electrode site (all *P* > 0.05). *Post hoc* simple effects analysis revealed no significant difference in beta (100–500 ms) energy values between the experimental group pre-test and the control group pre-test (*P* > 0.05), confirming the initial homogeneity among participants. Significant differences emerged between the experimental group mid-test and the control group mid-test (*P* < 0.05), and between the experimental group post-test and the control group post-test (*P* < 0.001), indicating that beta (100–500 ms) energy values in the experimental group were markedly higher than those in the control group after the aerobic exercise intervention at both 6 and 12 weeks. Within the experimental group, beta (100–500 ms) energy values varied significantly across the three time points (*P* < 0.001), with mid-test (*P* < 0.01) and post-test (*P* < 0.001) values being significantly higher than pre-test values, and post-test values being higher than mid-test values (*P* < 0.05). In contrast, the control group exhibited no significant changes in beta (100–500 ms) energy values across the three time points (*P* > 0.05).

**TABLE 6:** Descriptive statistics and repeated measures ANOVA of beta (100∼500 ms) wave energy values at three time points under the Nogo condition M(SD).

Time points	Group	Electrode site	Energy value/dB	F	*P*
Pre-test	Control group (*n* = 23)	F3	−2.73 (0.41)	–	–
		Fz	−3.61 (1.04)	–	–
		F4	−1.79 (0.62)	–	–
	Experimental group (*n* = 20)	F3	−2.81 (0.35)	–	–
		Fz	−3.73 (0.35)	–	–
		F4	−1.85 (0.43)	–	–
Mid-test	Control group (*n* = 23)	F3	−2.68 (0.21)	–	–
		Fz	−3.55 (1.02)	–	–
		F4	−1.77 (0.54)	–	–
	Experimental group (*n* = 20)	F3	−2.30 (0.25)^*^^##^	–	–
		Fz	−3.21 (0.62)^*^^##^	–	–
		F4	−1.52 (0.35)^#^	–	–
Post-test	Control group (*n* = 23)	F3	−2.61 (0.18)	–	–
		Fz	−3.48 (0.22)	–	–
		F4	−1.70 (0.54)	–	–
	Experimental group (*n* = 20)	F3	−1.91 (0.54)^***^^###^^▲^	–	–
		Fz	−2.70 (0.72)^***^^##^^▲^	–	–
		F4	−1.22 (0.39)^**^^###^	–	–
Main effect of time point				11.52	< 0.001
Main effect of group				50.26	< 0.001
Main effect of electrode site				9.78	< 0.001
Interaction effect of time point × group				8.52	< 0.01
Interaction effect of time point × electrode site				0.83	0.37
Interaction effect of group × electrode site				1.30	0.24
Three-way interaction effect of time point × group × electrode site				0.91	0.35

For intergroup comparisons, statistical significance was determined by comparing with the control group, denoted as *P < 0.05 for mild significance, **P < 0.01 for moderate significance, and ***P < 0.001 for high significance. For intragroup comparisons, statistical significance was assessed by comparing with pre-test results, denoted as #*P* < 0.05 for mild significance, ##*P* < 0.01 for moderate significance, and ###*P* < 0.001 for high significance; and by comparing with mid-test results, denoted as ▲*P* < 0.05 for mild significance.

The diagram presents the beta band energy values in the 100–500 ms time window at three electrode sites (Fz, F3, F4) for the control group and the experimental group at the pre-test, mid-test, and post-test. The bars represent the energy values in dB. It can be clearly seen that there are significant differences in beta band energy values among different electrode sites and between the groups at different time points, with the experimental group showing higher energy values especially after the aerobic exercise intervention.

**FIGURE 6:**
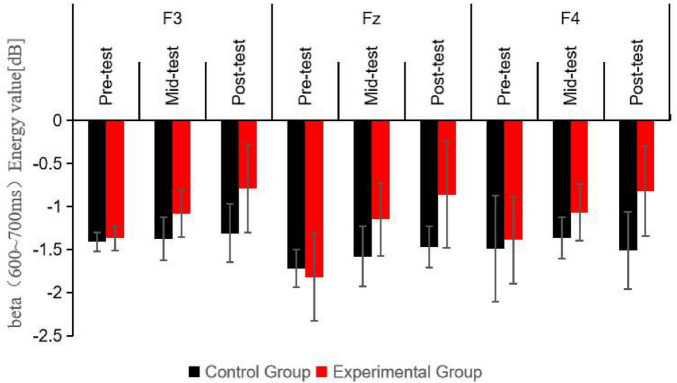
Schematic diagram comparing beta wave (600∼750 ms) energy values at different electrode sites for participants under the Nogo condition at three time points.

A repeated measures ANOVA was conducted to evaluate the beta band energy values (600–750 ms) for the experimental and control groups across three time points (pre-test, mid-test, post-test), two groups (control and experimental), and three electrode sites (Fz, F3, F4). The results, as detailed in Table 7 and depicted in Figure 6, demonstrated significant main effects for time point [*F*_(2, 123_) = 11.06, *P* < 0.001, *η^2^* = 0.37] and group [*F*_(1, 123)_ = 39.34, *P* < 0.001, *η^2^* = 0.52], while the main effect for electrode site was not significant (*P* > 0.05). A significant interaction was observed between time point and group [*F*_(2, 123)_ = 7.52, *P* < 0.01, *η^2^* = 0.19], yet interactions between time point and electrode site, group and electrode site, and the three-way interaction of time point, group, and electrode site were all non-significant (*P* > 0.05).

**TABLE 7:** Descriptive statistics and repeated measures ANOVA of beta (600∼750 ms) wave energy values at three time points under the Nogo condition M(SD).

Time points	Group	Electrode site	Energy value/dB	F	*P*
Pre-test	Control group (*n* = 23)	F3	−1.41 (0.11)	–	–
		Fz	−1.72 (0.22)	–	–
		F4	−1.49 (0.62)	–	–
	Experimental group (*n* = 20)	F3	−1.37 (0.14)	–	–
		Fz	−1.82 (0.51)	–	–
		F4	−1.39 (0.51)	–	–
Mid-test	Control group (*n* = 23)	F3	−1.38 (0.25)	–	–
		Fz	−1.58 (0.35)	–	–
		F4	−1.37 (0.24)	–	–
	Experimental group (*n* = 20)	F3	−1.08 (0.27)*	–	–
		Fz	−1.15 (0.42)^*^^##^	–	–
		F4	−1.07 (0.33)^*^^#^	–	–
Post-test	Control group (*n* = 23)	F3	−1.31 (0.34)	–	–
		Fz	−1.47 (0.24)	–	–
		F4	−1.51 (0.45)	–	–
	Experimental group (*n* = 20)	F3	−0.79 (0.51)^**^^###^^▲^	–	–
		Fz	−0.86 (0.62)^***^^###^^▲^	–	–
		F4	−0.82 (0.52)^***^^##^	–	–
Main effect of time point				11.06	< 0.001
Main effect of group				39.34	< 0.001
Main effect of electrode site				1.92	0.09
Interaction effect of time point × group				7.52	< 0.01
Interaction effect of time point × electrode site				1.25	0.31
Interaction effect of group × electrode site				1.43	0.25
Three-way interaction effect of time point × group × electrode site				1.24	0.33

For intergroup comparisons, statistical significance was determined by comparing with the control group, denoted as *P < 0.05 for mild significance, **P < 0.01 for moderate significance, and ***P < 0.001 for high significance. For intragroup comparisons, statistical significance was assessed by comparing with pre-test results, denoted as #*P* < 0.05 for mild significance, ##*P* < 0.01 for moderate significance, and ###*P* < 0.001 for high significance; and by comparing with mid-test results, denoted as ▲*P* < 0.05 for mild significance.

*Post hoc* simple effects analysis indicated no significant difference in beta (600–750 ms) energy values at the pre-test between the experimental and control groups (*P* > 0.05), confirming the initial homogeneity among participants. Significant differences were found between the experimental group mid-test and the control group mid-test (*P* < 0.05), and between the experimental group post-test and the control group post-test (*P* < 0.001), suggesting that the beta (600–750 ms) energy values in the experimental group were markedly higher than those in the control group following 6 and 12 weeks of aerobic exercise intervention. Within the experimental group, beta (600–750 ms) energy values varied significantly across the three time points (*P* < 0.001), with mid-test (*P* < 0.05) and post-test (*P* < 0.001) values being significantly higher than pre-test values, and post-test values being higher than mid-test values (*P* < 0.05). In contrast, the control group exhibited no significant changes in beta (600–750 ms) energy values across the three time points (*P* > 0.05).

This diagram shows the beta band energy values at different electrode sites for the control and experimental groups during the Nogo trials in the 600–750 ms time interval. The bars represent the energy values in dB. It can be seen that there are also significant differences between the groups and changes over time in this time window, with the experimental group generally having higher energy values after the aerobic exercise intervention, which further indicates the impact of exercise on the neural activity related to the Nogo task in this specific time and frequency range.

## 4 Discussion

### 4.1 Discussion of behavioral outcomes

The influence of exercise duration on the enhancement of inhibitory control through aerobic exercise is a critical factor warranting investigation. There is a lack of research, both domestically and internationally, on how varying durations of aerobic exercise impact inhibitory control in college students with IA. Our longitudinal study aimed to address this gap by examining the effects of moderate-intensity aerobic exercise on the inhibitory control of this specific demographic and tracking changes over time. The simple effects analysis revealed that the Nogo accuracy rate in the experimental group significantly improved after both 6 and 12 weeks of moderate-intensity aerobic exercise compared to the control group. Furthermore, within the experimental group, the Nogo accuracy rate also showed significant improvement after 6 and 12 weeks of exercise, with a more pronounced increase observed after 12 weeks. These findings suggest that moderate-intensity aerobic exercise can enhance inhibitory control in college students with IA, with the improvement becoming more pronounced as the duration of the exercise intervention increases. A recent meta-analysis supports our findings, indicating that long-term aerobic exercise significantly enhances inhibitory control in healthy children (Zang et al., 2024). Some researchers suggest that Go and Nogo tasks reflect the brain’s activation and inhibition functions, respectively (Gao et al., 2022). Our study’s results indicate that 12 weeks of moderate-intensity aerobic exercise selectively improved the Nogo task accuracy rate among college students with IA, without affecting the Go task. This suggests that moderate-intensity aerobic exercise may have a more significant impact on the reactive inhibition function of college students with IA.

### 4.2 Discussion of time-frequency results

Our investigation into the effects of moderate-intensity aerobic exercise on inhibitory control among college students with IA incorporated both behavioral metrics and ERP time-frequency analysis. The ERP time-frequency analysis revealed that, compared to the control group, the beta band oscillatory power during Nogo trials was significantly increased after both 6 and 12 weeks of moderate-intensity aerobic exercise, with a more pronounced increase observed after 12 weeks. These findings suggest that moderate-intensity aerobic exercise enhances beta band energy associated with the Nogo task in this demographic, and that this enhancement intensifies with prolonged exercise intervention durations.

Moreover, the study revealed that beta energy values at the midline (Fz) were significantly higher than those at the lateral sites (F3, F4). This suggests that frontal midline beta band oscillations play a crucial role in inhibitory functions, potentially due to the functional specificity of distinct brain regions. The Fz region may be critical in integrating information from various brain areas to exert precise control over behavioral responses during inhibitory tasks (Miller and Cohen, 2001). In contrast, while F3 and F4 are also involved in cognitive processing, their roles tend to be more specialized and focused on specific aspects of information processing (Shallice and Cooper, 2011).

The functional interpretation of beta band energy values under Nogo stimulation remains a contentious issue. Some researchers suggest that an increase in beta band energy under Nogo stimulation may reflect the motor inhibition process. Specifically, when participants identify a Nogo stimulus and successfully suppress an impending motor response, this inhibition is believed to manifest as an increase in beta band energy in the brain’s electrical activity (Chen et al., 2020; Gajewska-Dendek et al., 2019; Hwang et al., 2014). Other scholars propose that beta band activity is linked to attentional control and the mechanism for updating adaptive responses based on the current environment. During the processing of Nogo stimuli, adjustments in attentional resources are necessary to address new situations, and these adjustments are reflected in changes in beta band energy values (Wu et al., 2019). Additionally, it has been suggested that increased beta band energy values are associated with the brain’s preparation and adaptation to anticipated future events. In the anticipatory phase, the brain is thought to enhance its readiness for forthcoming stimuli by increasing beta band energy (Schmiedt-Fehr et al., 2016). Our study indicates that the discrepancies in interpreting the beta band’s function may be related to the selection of time windows. In the initial 100–500 ms following Nogo stimulation, information recognition and judgment predominate, with beta band energy values primarily reflecting inhibitory control functions. After 600–750 ms, once the motor response has subsided, beta band energy values may indicate preparation and adaptation for subsequent stimuli. Our findings reveal that after aerobic exercise, the 13–20 Hz beta band energy in the frontal lobe region, under Nogo stimulation, significantly increased within both the 100–500 and 600–750 ms time windows. This suggests that moderate-intensity aerobic exercise can enhance the inhibitory control capabilities of college students with IA and allocate additional resources for anticipating and adapting to subsequent stimuli. Miller et al. have demonstrated that beta band energy in the prefrontal cortex is associated with top-down processing and inhibition (Hwang et al., 2014; Miller et al., 2018; Picazio et al., 2014). Our results imply that moderate-intensity aerobic exercise can enhance college students’ capacity to prepare for and adapt to subsequent stimuli. Given that inhibitory responses require sufficient adaptive preparation, the observed increase in beta band energy values following aerobic exercise may explain the improved accuracy in responding to Nogo stimuli. This suggests that increasing beta band energy through aerobic exercise intervention can improve cognitive control, particularly in contexts that demand inhibitory responses.

During physical activity, the human body experiences an increased need for inhibitory control compared to a resting state. This requirement involves not only managing limb movements and athletic performance but also continuously adjusting psychological states in accordance with the exercise intensity. Such adjustments play a role in promoting the enhancement of inhibitory control to a certain degree.

In the context of aerobic exercise intervention for university students with IA, the improvement in inhibitory control can be explained by the relationship between exercise and neural impulse conduction. According to segmental control mechanisms and feedback systems (Corcos et al., 2022; Kruk, 2022), higher cortical centers regulate the activities of the brainstem and spinal cord through descending neural impulses. This regulation affects the efficiency of brainstem pathway transmission. Specifically, the descending neural impulses from higher cortical centers can directly act on motor neurons in the brainstem and spinal cord (pyramidal system) and indirectly influence motor functions through various efferent pathways originating from the brainstem (extrapyramidal system) (Kandel et al., 2000). At the same time, sensory information from muscles, joints, skin, and other sensory organs is transmitted back to higher cortical centers (Hayward, 1997; Wundt, 1999). The interaction between these control mechanisms and feedback systems enables precise motor control and sensory processing.

Previous empirical studies have highlighted the crucial role of neural impulse transmission efficiency in optimizing the functions of the central nervous system (CNS), especially in strengthening inhibitory control within the brain (Ikeda and Shibasaki, 1992; Ikeda et al., 1992; Neshige et al., 1988). Aerobic exercise is associated with an elevated frequency of neural impulses between higher cortical centers and motor effectors, which leads to enhanced neural transmission efficiency. This is in line with Pavlovian theory, which posits that repeated neural stimulation within central pathways can facilitate impulse conduction (Pavlov, 1960). As a result, this contributes to the improvement of inhibitory control mechanisms and the overall enhancement of CNS performance.

Our longitudinal observation of college students with IA throughout the intervention period has disclosed a notable trend: the enhancement effect on beta band energy values intensifies with the extended duration of the exercise intervention. This correlation may be attributed to the temporal dynamics through which aerobic exercise bolsters brain plasticity, particularly in individuals with IA. Empirical studies have established a robust link between gray matter density and executive functions, which encompass the capacity for inhibitory control. Functional near-infrared spectroscopy (fNIRS) has revealed that adolescents who engage in long-term regular exercise display increased gray matter density in the bilateral thalamus and left premotor cortex, contrasting with those with limited or no exercise engagement or those subjected to brief exercise interventions (Jalanko et al., 2024; Salvan et al., 2021; Yuan et al., 2020). Building on these findings, our study posits that an increment in exercise duration may lead to a concomitant rise in gray matter density within the bilateral thalamus and left premotor cortex of college students with IA, thereby enhancing their inhibitory control. Further validation of this hypothesis will necessitate additional neuroimaging studies.

In this study, we also conducted preliminary monitoring of other brain waves. The results showed that, except for beta waves, under the experimental conditions related to this study, other brain waves (such as gamma waves, etc.) did not exhibit obvious change trends among different measurement time points, electrode sites, as well as between the experimental group and the control group. This result further supports the rationality of focusing on beta waves in this study, indicating that beta waves may be key neurophysiological indicators for exploring the impact of aerobic exercise on the inhibitory control in college students with internet addiction. However, this does not mean that other brain waves will not play important roles in other research contexts or for different groups. Future research can explore this in depth.

## 5 Conclusion

This study employed the Go/Nogo task, combined with ERP technology and time-frequency analysis, to investigate the impact of moderate-intensity aerobic exercise on inhibitory control in college students with IA and its temporal characteristics. The findings highlight the following:

1. Moderate-intensity aerobic exercise significantly enhances inhibitory control in college students with IA, with the magnitude of this enhancement increasing as the exercise intervention duration extends.
2. This form of exercise reduces the temporal latency required for early conflict monitoring within the reactive inhibition framework and decreases the cognitive load associated with the active inhibition process, thereby enhancing participants’ adaptive preparatory responses to imminent Nogo stimuli.

In summary, our study concludes that the increase in beta band energy is a critical neurophysiological marker, indicating the mechanisms by which moderate-intensity aerobic exercise enhances inhibitory control in college students with IA.

## 6 Limitations and future perspectives

The present study, while providing valuable insights, has certain limitations and opens avenues for future research:

1. The study focused on male university students with IA. Given that EEG data can be influenced by variables such as age and gender, as indicated by previous research, future studies should explore whether the findings are applicable to female university students with IA.
2. Participant physical activity levels were assessed using the IPAQ. The lack of accelerometer-based continuous 24 h measurements over 7 days may have affected the precision of our results. Future studies should use more precise measurement techniques to determine participant physical activity levels.
3. This study examined the neural mechanisms of aerobic exercise on executive function in university students with IA from an electrophysiological perspective alone. Future research could benefit from incorporating additional physiological markers, such as near-infrared spectroscopy (NIRS) and functional magnetic resonance imaging (fMRI), to further elucidate the neural mechanisms through which aerobic exercise enhances executive function in this population.
4. The study’s sample size was relatively small, which may limit the generalizability and statistical power of the findings. Although the necessary minimum sample size was determined via power analysis during the study design, and efforts were made to meet this target in recruitment, the final analysis included a limited number of participants. A smaller sample size may not fully capture the diversity of the broader college student population with IA, potentially introducing biases into the study outcomes.
5. It should be noted that the current study primarily focused on the frontal regions, particularly the changes in beta band energy at the midline frontal Fz, left prefrontal F3, and right prefrontal F4. However, the inhibitory network is much broader than just the prefrontal areas. Previous studies have shown that it also includes frontal motor areas (Coxon et al., 2006; Mattia et al., 2012), parietal areas (Cai et al., 2016; Swann et al., 2009), and basal ganglia (Mancini et al., 2019; Redgrave et al., 2010). Therefore, our research, limited to the frontal regions, represents a certain limitation, and future studies could expand the scope of investigation to cover a more comprehensive inhibitory network.

## Data Availability

The original contributions presented in this study are included in this article/supplementary material, further inquiries can be directed to the corresponding author.
